# Trends in malaria morbidity following the introduction of artesunate plus amodiaquine combination in M'lomp village dispensary, south-western Senegal

**DOI:** 10.1186/1475-2875-7-215

**Published:** 2008-10-24

**Authors:** Sophie Sarrassat, Paul Senghor, Jean Yves Le Hesran

**Affiliations:** 1 Unité de Recherche, Santé de la mère et de l'enfant en milieu tropical, IRD, UR010, Université Pairs Descartes, Faculté de Pharmacie, Paris, France; 2 Unité de Service, Espace de recherche intégrée sur la santé des populations, IRD, US09, Dakar, BP 1386, Sénégal

## Abstract

**Background:**

In Thailand, South Africa and Zanzibar, a decrease in malaria morbidity was observed following the introduction of artemisinin-based combination therapy (ACT). In Senegal, therapeutic trials supervised the in vivo efficacy of artesunate plus amodiaquine from 1999 to 2005 at the M'lomp village dispensary. The trends in malaria morbidity in this village were evaluated from 2000 to 2002.

**Methods:**

Each year, between July and December inclusive, fevers treated with antimalarials and slide-proven, uncomplicated malaria cases were collected from dispensary health records. Data were also collected in 1998, just prior to ACT introduction. Pearson's chi square tests and Student tests were used to compare two percentages or two means respectively (α = 0.05).

**Results:**

Between 1998 and 2002, the total number of fevers treated with antimalarials and their repetitiveness progressively decreased: From 2824 to 945 fevers and from 17.6% to 9.7% (RR_1998–2002 _= 0.55; [0.44–0.69]; p < 0.0001) respectively. Considering uncomplicated malaria cases only, a decrease was observed in their total number between 2001 and 2002, from 953 to 570 cases. The incidence rate and repetitiveness also decreased. The incidence rate fell from 46.1% in 2001 to 37.5% in 2002 (p < 0.0001) and the repetitiveness decreased from 13.0% in 2000 to 6.6% in 2002 (RR_2000–2002 _= 0.51; [0.35–0.72]; p = 0.0001).

**Conclusion:**

The percentage of uncomplicated malaria cases treated with ACT increased, from 18.9% in 2000 to 64.0% in 2002, making it tempting to conclude an impact on malaria morbidity. Nonetheless, the decline in incidence rate of uncomplicated malaria was slight and a lower recorded rainfall was reported in 2002 which could also explain this decline. The context in which ACT is introduced affects the impact on malaria morbidity. In M'lomp, in contrast to studies in Thailand, South Africa and Zanzibar, ACT coverage of malaria cases was low and no vector control measure was deployed. Moreover, the malaria transmission level is higher. In sub-Saharan countries, in order to optimize the impact on malaria morbidity, ACT deployment must be supported, on the one hand, by a strengthening of public health system to ensure a high ACT coverage and, on the other hand, by others measures, such vector control measures.

## Background

Since 2001, in the face of the extension of *Plasmodium falciparum *resistance to commonly used antimalarials, the WHO has advocated the adoption of artemisinin-based combination therapy (ACT) for treating uncomplicated malaria [1]. The use of ACT is motivated firstly by its high efficacy [2,3]. Moreover, the combination of two antimalarials, with independent modes of action reduces the risk of emergence of drug resistances [4]. Lastly, artemisinin derivates reduce gametocyte carriage and could, therefore, decrease malaria transmission and morbidity [5,6]. In camps for displaced persons on the north-western border of Thailand, a 67% reduction in the incidence of malaria was observed in 1997, following of four years use of artesunate plus mefloquine (AS+MQ) [7]. In the north-western Tak province of Thailand, a 60% reduction in the incidence of malaria was reported in 2003, two years following the introduction of AS+MQ [8]. In KwaZulu-Natal province of South Africa, the total annual number of outpatient malaria cases at four health facilities fell by nearly 100% in 2003, three years after the deployment of artemether/lumefantrine (AM/LUM) [9]. Lastly, in North A District of Zanzibar, the total annual number of outpatient malaria diagnoses decreased by 67% in children under five years old in 2005, two years after the implementation of artesunate plus amodiaquine (AS+AQ) [10].

In Senegal, since March 2006, the AS+AQ combination has been recommended for treating uncomplicated malaria [11]. From 1999 to 2005, therapeutic trials supervised the in vivo efficacy and safety of AS+AQ at four health facilities in the Oussouye department, particularly at the M'lomp village dispensary [12,13]. The trends in malaria morbidity in this village were evaluated from 2000 to 2002 and the possible impact of the introduction of ACT on these trends was discussed.

## Methods

### Study site

M'lomp is a village located on the mouth of the Casamance River in south-western Senegal. The climate is characterized by the alternation of two seasons, a rainy season, from June to October, and a dry season, from November to May. On January first 2000, 8 000 inhabitants were living in M'lomp [14]. The dispensary is able to stain and read thick blood smears. Malaria is mesoendemic with the majority of cases occurring between July and December. In November 1994, the parasite prevalence was 51% in children less than 15 years old [15]. *P. falciparum *is the predominant malaria specie and *Anopheles gambiae *complex is considered the main vector. The entomological inoculation rate (EIR) was calculated to be 30 infected bites per person-year [16]. The first in vivo chloroquine-resistant cases were detected in 1990 [15]. During the rainy seasons 1996 and 1997, the parasitological failure rate by day 14 was as high as 76% [17].

### Introduction of ACT at the M'lomp dispensary

In 1999, a comparative therapeutic trial of AS+AQ versus AQ was carried out to assess the in vivo efficacy and safety of ACT. From 2000 to 2005, non-comparative trials with the aim of supervising the efficacy of ACT were conducted. The detailed protocols used in these trials can be found in Adjuik *et al *and Brasseur *et al *[12,13]. All patients treated with ACT had slide positive *P. falciparum *uncomplicated malaria. The dose regimen was 4 mg/kg of AS and 10 mg/kg of AQ base per day for three days. All the doses were administered under clinical supervision. Non-eligible patients were treated with intramuscular injections of quinine (QN IM) followed by oral chloroquine (CQ) according to the usual practice of the dispensary.

### Assessment of the malaria morbidity in M'lomp

Malaria morbidity was assessed from July to December inclusive, in 2000, 2001 and 2002, by an observational retrospective study on fevers and uncomplicated malaria cases treated with antimalarials at the dispensary. Data were also collected in 1998, just prior to ACT introduction. Data from the year 1999 were not considered in the study because only children less than 10 years old were treated with ACT, while from 2000 to 2002 all patients, independent of their age, were treated. In 2000 and 2001, ACT was administered for three months each year, from August to October. In 2002, ACT was administered for five months, from August to December.

### Collecting of malaria morbidity indicators

Two indicators of malaria morbidity were chosen: uncomplicated malaria cases and fevers treated with antimalarials. Uncomplicated malaria cases were defined as febrile patients with a positive thick blood smear. Fevers treated with antimalarials included uncomplicated malaria cases and presumed malarial fevers, diagnosed clinically by the nurse. Data were collected from dispensary health records. Name, surname, age, sex and address were collected to identify subjects. Dates of consultation, results of thick blood smear and antimalarials prescribed were also collected. Fevers with negative thick blood smear were sometimes treated with antimalarials. These fevers were excluded from the study. Furthermore, recorded rainfalls were collected from the Departmental Service of Rural Development of Oussouye, located 9 km from M'lomp.

### Measuring repetitiveness of consultation

Repetitiveness was defined as the frequency of consultation at the dispensary by one subject. It was studied for each malaria morbidity indicator. Subjects were identified by their full name, sex, age and address. Patients living outside of the dispensary's area were excluded from the study because they were less likely to return for a consultation at the dispensary. Lastly, an investigator coming from the village and acquainted with the inhabitants validated the identification of subjects.

### Statistical analysis

Between July and December inclusive in 2000, 2001 and 2002, for each malaria morbidity indicator the distribution of prescribed antimalarial treatments, the total number of cases and their repetitiveness in subjects were measured. Uncomplicated malaria incidence rates were calculated as the percentage of positive thick blood smears. Between July and December inclusive in 1998, the frequency of thick blood smears in cases of fevers was less than in 2000–2002, 42.9% in 1998 compared to 74.0% in 2000, 76.7% in 2001 and 80.2% in 2002. Because of this, the total number of uncomplicated malaria cases and their repetitiveness in subjects were not studied in 1998.

The analysis of the repetitiveness for each malaria morbidity indicator consisted of the calculation of the mean number of consultations between July and December inclusive per subject and the calculation of the percentage of subjects consulting a second time or more relative to the total number of subjects having consulted one time. Moreover, the relative risk for one subject to consult a second time for fever treated with antimalarials between July and December inclusive in 2002 was calculated relative to the same period in 1998. For uncomplicated malaria, the relative risk was calculated between July and December inclusive in 2002 relative to the same period in 2000. Lastly, the time delays between the first and second consultation were calculated and the mean time delays were compared from 1998 to 2002 for fever treated with antimalarials and from 2000 to 2002 for uncomplicated malaria.

Statistical analyses were performed using Stata version 6.0. Confidence intervals of percentages and relative risks were given at 95%. Pearson's chi square tests and Student tests were used to compare two percentages or two means respectively (α = 0.05).

## Results

### Distribution of antimalarial treatments prescribed by the dispensary

In 1998, 94.4% of fevers and 100% of uncomplicated malaria cases were treated with QN IM followed by oral CQ (Table 1). From 2000 to 2002, the percentage of uncomplicated malaria cases treated with ACT increased, from 18.9% in 2000 to 64.0% in 2002. The prescription of CQ or AQ as monotherapy was rare.

**Table 1:** Distribution of antimalarial treatments prescribed by the dispensary

		**Total (n)**	**QN IM (n, %)**		**AS+AQ (n, %)**		**CQ or AQ (n, %)**	
**1998**	**Fevers treated with antimalarials**	**2 824**	2 666	**94.4**	0	**0**	158	**5.6**
	**Uncomplicated malaria cases**	**599**	599	**100**	0	**0**	0	**0**
								
**2000**	**Fevers treated with antimalarials**	**2 058**	1 696	**82.4**	217	**10.5**	145	**7.0**
	**Uncomplicated malaria cases**	**1 147**	877	**76.5**	217	**18.9**	53	**4.6**
								
**2001**	**Fevers treated with antimalarials**	**1 582**	1 191	**75.3**	305	**19.3**	86	**5.4**
	**Uncomplicated malaria cases**	**953**	601	**63.1**	305	**32.0**	47	**4.9**
								
**2002**	**Fevers treated with antimalarials**	**945**	550	**58.2**	365	**38.6**	30	**3.2**
	**Uncomplicated malaria cases**	**570**	205	**36.0**	365	**64.0**	0	**0**

QN IM: intramuscular injections of Quinine followed by oral Chloroquine (CQ); AS+AQ: Artésunate plus Amodiaquine

### Total number of fevers treated with antimalarials and uncomplicated malaria cases

**Figure 1:**
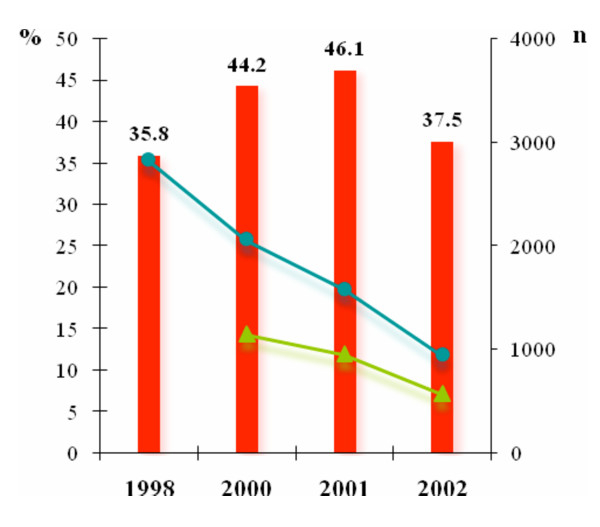
**Total number of fevers treated with antimalarials – Total number and incidence rate of uncomplicated malaria cases**. Blue and green lines represent the total number of fever treated with antimalarials (n) and the total number of uncomplicated malaria cases (n) respectively. Red bars represent the incidence rate of uncomplicated malaria (%) (Values are indicated).

In 1998, 2000 and 2001, the total number of consultations at the dispensary with any complaint was relatively stable: 8327, 7868 and 7322 consultations respectively. In 2002, this fell by 36% from the previous year to 4707 consultants. The total number of fevers treated with antimalarials decreased progressively: From 2824 in 1998 to 2058 in 2000 then 1582 in 2001 and 945 in 2002, corresponding to a 67% reduction between 1998 and 2002 (Figure 1). The total number of uncomplicated malaria cases was relatively constant in 2000 and 2001 with 1147 cases and 953 cases respectively. In 2002, it fell by 40% from the previous year to 570 cases.

### Incidence rate of uncomplicated malaria

The incidence rate of uncomplicated malaria was 35.8% in 1998. It increased significantly to 44.2% in 2000 (p < 0.0001) and was not significantly different in 2001, 46.1% (p = 0.23). In 2002, the rate declined to 37.5%, corresponding to a significant 19% reduction (p < 0.0001) (Figure 1).

### Monthly incidence rates of uncomplicated malaria

**Figure 2:**
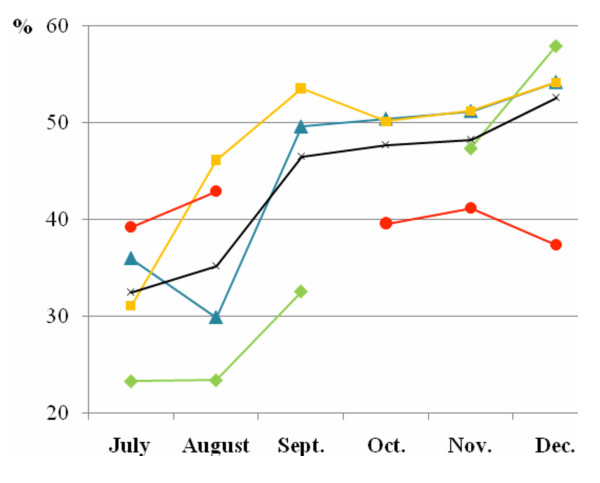
**Monthly incidence rates of uncomplicated malaria**. Green, blue, yellow and red lines represent the monthly incidence rates of uncomplicated malaria (%) between July and December in 1998 (October excluded), 2000, 2001 and 2002 (September excluded) respectively. The black line represents the mean monthly incidence rates of uncomplicated malaria (%) from 1998 to 2002 (1999 excluded).

Because of the very limited number of thick blood smears made in October 1998 and anomalous incidence rates for September 2002, these two rates were excluded from the study (Figure 2). From March to June each year, incidence rates were at a minimum, between 20% and 30%. From July, the incidence rate increased progressively to a maximum at the end of the year. In 1998, the rates from July to September were lower (between 23.3% and 32.6%), than in 2000, 2001 and 2002 (between 31.1% and 53.6%). From October to December in 1998, 2000 and 2001, the rates were at a maximum, varying between 50.2% and 58.0%. However, in 2002, rates lower than in three previous years were observed, 39.6% in October, 41.2% in November and 37.4% in December. Compared to the same months in 2001, differences were significant (p ≤ 0.04).

### Repetitiveness of fevers treated with antimalarials

In 1998 and 2000, the mean number of fevers treated with antimalarials per subject was similar, 1.20 and 1.18 respectively. In 2001 and 2002, it decreased to 1.14 and 1.11 respectively.

The percentage of subjects consulting a second time also decreased: From 17.6% in 1998 to 9.7% in 2002, corresponding to a significant 45% reduction in the risk to consult a second time (RR_1998–2002 _= 0.55; [0.44–0.69]; p < 0.0001) (Table 2). Whatever the year, less than 2.5% of subjects consulted three times.

**Table 2:** Repetitiveness of fevers treated with antimalarials and uncomplicated malaria cases

	**Fevers treated with antimalarials**			**Uncomplicated malaria cases**		
	**C1**	**C2**	**C3**	**C1**	**C2**	**C3**
**1998**	**2 346**	**413**	**55**	**-**	-	-
		17.6; [16.1–19.2]	2.3; [1.8–3.0]		-	-
**2000**	**1 750**	**261**	**38**	**1 000**	**130**	**15**
		14.9; [13.3–16.7]	2.2; [1.5–3.0]		13.0; [11.0–15.2]	1.5; [0.8–2.5]
**2001**	**1 383**	**161**	**34**	**856**	**80**	**16**
		11.6; [10.0–13.4]	2.5; [1.7–3.4]		9.3; [7.5–11.5]	1.9; [1.1–3.0]
**2002**	**854**	**83**	**7**	**533**	**35**	**2**
		9.7; [7.8–11.9]	0.8; [0.3–1.7]		6.6; [4.6–9.0]	0.4; [0.05–1.3]

C1: Total of subjects having consulted at least one time

C2 and C3: Total and percentage (IC_95%_) of subjects consulting two and three times

### Repetitiveness of uncomplicated malaria cases

From 2000 to 2002, the mean number of uncomplicated malaria cases per subject decreased progressively: From 1.15 to 1.11 then 1.07 cases respectively. The percentage of subjects consulting a second time also decreased: From 13.0% in 2000 to 6.6% in 2002, corresponding to a significative 49% reduction in the risk to consult a second time (RR_2000–2002 _= 0.51; [0.35–0.72]; p = 0.0001) (Table 2). Whatever the year, less than 1.9% of subjects consulted three times.

### Time delays between the first and second consultation

From 1998 to 2002, the mean time delay between the first and second consultation for fever treated with antimalarials changed from 64 ± 38 days to 58 ± 37 days (p = 0.22). From the first to the second uncomplicated malaria case, the mean time delay increased from 54 ± 36 days in 2000 to 65 ± 45 days in 2002. However this difference was not significant (p = 0.13).

### Recorded rainfall from 1998 to 2002

From 1998 to 2001, the recorded rainfall increased progressively: From 1233 mm in 1998 to 1365 mm in 2000 and 1502 mm in 2001. In 2002, the recorded rainfall decreased to 1062 mm. The years 1998 and 2002 had lower recorded rainfalls in June and July in comparison with the years 2000 and 2001.

## Discussion

The purpose of this study was to investigate the trend in malaria morbidity in M'lomp, from 2000 to 2002, during three years of AS+AQ utilisation. A second objective was to discuss a possible impact of ACT introduction on malaria morbidity.

### The trend in malaria morbidity and ACT introduction in M'lomp

Between 1998 and 2002, both the total number of fevers treated with antimalarials and their repetitiveness progressively decreased. Considering uncomplicated malaria cases only, a decrease was observed in their total number and incidence rate between 2001 and 2002. Their repetitiveness decreased between 2000 and 2002. Furthermore, the results of non-comparative trials carried out from 2000 to 2005 showed high clinical and parasitological cure rates at day 28, ranging from 88.5% to 96.7% [13].

The percentage of uncomplicated malaria cases treated with ACT increased from 2000 to 2002 making it tempting to conclude an impact on malaria morbidity. However, a lower recorded rainfall was observed in 2002, which could also explain this decline. In 1998, prior to the introduction of ACT, the recorded rainfall was also low and the incidence rate of uncomplicated malaria was lower than in 2000 and 2001, years of high rainfalls. Nonetheless, it is difficult to determine the impact that a variation in rainfall has on malaria morbidity, especially since the rainfall was always high, more than one meter in five months. Moreover, the relationship between rainfall and transmission is also influenced by rhythm and intensity of the rain [18].

Even if the decline in the incidence rate of uncomplicated malaria in 2002 was due to ACT administration, this was, despite its statistical significance, small. Furthermore, the dispensary frequentation fell by 36% between 2001 and 2002, while during the same time the proportion of consultations due to uncomplicated malaria remained similar at 13% and 12% respectively. During the transmission season, malaria constitutes between 30% and 50% of fever consultations. The remaining 50 to 70% of fevers resulting in consultation are likely due to respiratory or gastrointestinal infections. In 2002, a decline in the incidence of fevers of non-malarial origin could therefore explain the diminution in the numbers of fevers treated with antimalarials. However, between 1998 and 2001, whilst the dispensary frequentation was relatively stable and rainfall had increased, the diminution of fevers treated with antimalarials had already started. This diminution could be explained by a reduction in the decision to treat presumptively. Indeed, from 2000, the presence of medical staff to conduct therapeutic trials might have encouraged the nurse to practice more thick blood smears and to refine his antimalarial prescriptions. This would fit with the observation that the frequency of blood smears in patients with fever increased from 42.9% in 1998 to 74.0% in 2000.

In conclusion, the impact of AS+AQ introduction at the M'lomp dispensary could not be established. Even though a decline in the malaria morbidity was observed, co-factors can also explain it. It is interesting to note the decrease in repetitiveness of uncomplicated malaria cases in 2001, whereas the recorded rainfall was high and the dispensary frequentation was similar to 2000. The repetitiveness is a longitudinal indicator. Its decrease in 2001, while the incidence rate did not change, well illustrates the supplementary information that it provided in comparison with a transversal indicator. This type of indicator should be used more frequently for studying the trends in malaria morbidity. In three different endemic areas, Trape *et al *showed the relation between the total number of attacks over an entire lifetime and the level of transmission [19].

### Impact of ACT in Thailand, South Africa and Zanzibar

The decrease in malaria morbidity observed in Thailand, South Africa and Zanzibar, has been attributed to early treatment of malaria cases with ACT [7-10]. In these three countries, it is certain that the much higher efficacy of ACT, in comparison to previously used antimalarials, did reduce the therapeutic failures and caused a rapid decrease in malaria morbidity. In camps for displaced persons in Thailand, therapeutic failure rates by day 28 in 1994 were 31% after a high dose of MQ compared to 2% following AS+MQ treatment [20]. In KwaZulu-Natal province, South Africa, parasitological failure rates by day 42 were 89% after SP treatment in 2000 in comparison to 1% after AM/LUM treatment in 2002 [9]. In North A District, Zanzibar, therapeutic failure rates among children under five years old were 60% by day 14 after CQ treatment in 2000 compared to 6% by day 28 after AS+AQ treatment in 2003 [10].

However, in South Africa, Barnes *et al *concluded that the impact of ACT could not be seen in the absence of indoor chemical-residual spraying, reintroduced to the area [9]. In Zanzibar, from February 2006, impregnated bed nets were distributed to populations at risk. In May 2006, a 94% further reduction of the *P. falciparum *prevalence was reported among children under five years old [10]. In Thailand, vector control measures were already implemented before ACT introduction and therefore did not contribute to the observed impact [7,8].

Furthermore, all authors agreed that high ACT coverage of malaria cases was essential to obtain the impact on morbidity and was made possible by high-performance public health systems, the gratuity of ACT and information campaigns conducted amongst populations [8-10]. The particular management of camps for displaced persons in Thailand allowed covering almost 100% of cases [7]. In the Thai population of Tak province, ACT coverage of 80% was obtained by a considerable strengthening of health structures [8]. South Africa and Zanzibar also have high-performance public health systems [9,10]. In KwaZulu-Natal province, 97% of interviewed subjects declared seeking treatment at public health facilities in the event of having fevers [9].

Lastly, all authors considered that the reduction in frequency and density of gametocyte carriage due to artemisinin derivates could have contributed to the decrease in malaria morbidity [5,6,9,10]. In camps for displaced persons in Thailand, the gametocyte prevalence was eleven times higher in refugees treated with MQ (29.1%) than in refugees treated with an artemisinin derivative (2.6%) [5]. In Tak province, the gametocyte prevalence was significantly lower amongst populations covered by ACT, between 0.1% and 1.2%, than in Burmese villagers living on the north-eastern border of Myanmar, between 3.1% and 10.2% (p < 0.001) [8]. In North A District, Zanzibar, the gametocyte prevalence among children less than 15 years old was reduced from 1.5% to 0.4% in 2005, two years after ACT introduction [10]. In Kwa-Zulu Natal province, South Africa, the gametocyte prevalence was evaluated at 57% after SP treatment in 2000 compared to 2% after ACT treatment in 2002 [9].

Nonetheless, it is difficult to determine the proportion attributable to the reduction of gametocyte carriage in the decrease in malaria morbidity. The use of CQ, which has no effect on gametocyte carriage, but is associated with high coverage and early treatment, led to a 50% decrease in the incidence of presumptive malaria cases between August 1985 and July 1987 in Katana province of Zaire [21]. In the Niakhar area of Senegal, a weekly prospective follow up study of a cohort of 566 children with free access to health care for three months in the malaria transmission season led, in spite of the high level of CQ resistance, to no return to the dispensary in children who had receive this treatment and only one case of severe malaria [22].

### Factors limiting the ACT impact in M'lomp

Even though a decline in the incidence rate of uncomplicated malaria was observed in M'lomp, it was slight and the relationship with ACT introduction could not be firmly established. When considering the studies in Thailand, South Africa and Zanzibar, it appears that ACT introduction context determines the impact on malaria morbidity.

In M'lomp, data collected from dispensary health records reported frequent usage of QN throughout the study period. From 2000 to 2004, *in vitro *susceptibility studies reported 84% and 90% of *P. falciparum *sensitive isolates to QN and artemisinin respectively [23]. Thus, the majority of patients were successfully treated by QN and ACT introduction did not reduce therapeutic failures in similar proportions to those observed in Thailand, South Africa and Zanzibar. It also important to note that, in M'lomp, no vector control measures were deployed during the study period. Moreover, the ACT coverage of malaria cases was low, particularly in 2000 and 2001. Furthermore, even if the dispensary is well frequented, an important number of fevers were probably treated at home. In sub-Saharan countries, self treatments are frequent and the level of care seeking at public health system is generally low. In Niakhar, Senegal, from 25% to 50% of fevers are not treated at the dispensary [24]. In the same way, migration of populations limited ACT coverage. In 1999, the census data from M'lomp showed that about 40% of inhabitants had been non-resident for a part of the year [14].

Lastly, the malaria transmission level is higher in M'lomp than in the north-western Thailand and KwaZulu-Natal province. The EIR was calculated to be 30 infected bites per person-year in M'lomp [16] compared to less than one infected bite per person-year in two other cited areas [6,8,9]. In Nord A District, Zanzibar, the gametocyte prevalence in 2003 was evaluated to be 13% among children from 6 to 14 years old [10], suggesting a lower malaria transmission level than in M'lomp. The study of the relationship between malaria transmission and morbidity showed that the impact of a transmission reduction on malaria morbidity is significantly higher when the initial transmission level is low. For EIR between 0.01 to 0.1 infective bites per person-year, the total number of malaria attacks over an entire lifetime appears directly proportional to the level of transmission and varies by a factor of ten, whereas for EIR ranged between 1 to 100, the total number of attacks over an entire lifetime, which is always very high, varies at most by a factor of two to three [19]. Furthermore, in areas of low transmission, malaria infections are predominantly symptomatic and can therefore be detected and treated with ACT [25]. In contrast, in high malaria transmission areas, asymptomatic infections are common and these semi-immune individuals act as a reservoir of gametocytes sustaining the transmission [26,27].

Finally, it is important to emphasize that in M'lomp ACT was administered in an experimental context, conditionally to a positive thick blood smear and with clinical supervision of drug intakes. This context allowed a full compliance of patients to ACT treatment, securing both schizonticide and gamétocytocide efficacies. In a context of routine use of ACT, patients' compliance may be incomplete. In the past, while monotherapy by CQ or SP were recommended, studies reported partial compliance [28,29]. The non-compliant patients, even though being clinically cured, will continue to host parasites and risk limiting the impact of ACT treatment on both malaria transmission and morbidity. In Zambia, where 61% of children less than five years were non-compliant to the ACT AS+SP [30], a study carried out on the same site and in children of the same age showed a low cure rate at day 28, 63%. In comparison, the day 28 cure rate in children for which all doses were clinically supervised was 84% [31]. To explain these low cure rates, the authors advanced the high level of resistance to SP and also suggested the impact of compliance on therapeutic efficacy.

## Conclusion

In M'lomp, even though a decline in the incidence rate of uncomplicated malaria was observed, it was slight and the impact of ACT introduction could not be clearly established. The context in which ACT is introduced affects the impact on malaria morbidity. In contrast to studies in Thailand, South Africa and Zanzibar, ACT coverage of malaria cases was low, no vector control measure was deployed and the level of malaria transmission is higher. In sub-Saharan countries, in order to optimize the impact on malaria morbidity, ACT deployment must be supported, on the one hand, by a strengthening of public health system to ensure a high ACT coverage and, on the other hand, by others measures, such vector control measures and interventions improving the compliance of patients to their treatment.

## Competing interests

The authors declare that they have no competing interests.

## Authors' contributions

SS collected data, carried out the analysis and interpretation of data and prepared the manuscript. PS helped to the identification of inhabitants of M'lomp in order to measure the repetitiveness. JYLH contributed in the analysis and interpretation of data and in the preparation of the manuscript. All authors read and approved the final manuscript.
